# A Study on Tannery Sludge as a Raw Material for Cement Mortar

**DOI:** 10.3390/ma12091562

**Published:** 2019-05-13

**Authors:** Jurgita Malaiškienė, Olga Kizinievič, Viktor Kizinievič

**Affiliations:** Laboratory of Composite Materials, Institute of Building Materials, Vilnius Gediminas Technical University, Linkmenų St. 28, LT 08217 Vilnius, Lithuania; olga.kizinievic@vgtu.lt (O.K.); viktor.kizinievic@vgtu.lt (V.K.)

**Keywords:** tannery sludge, waste, recycling, cement mortars, compressive strength, leaching

## Abstract

The paper analyses the properties (chemical and mineral composition, microstructure, density, etc.) of recycled tannery sludge (TS) and the possibilities for using it in cement mortar mixture. Mortar specimens containing 3–12% of tannery sludge by weight of cement and 3–9% of tannery sludge by weight of sand were tested. Flowability, density, ultrasonic pulse velocity (UPV), flexural and compressive strength, water absorption and sorptivity of the mortar were analysed. X-ray diffraction (XRD) and scanning electron microscopy (SEM) analysis of tannery sludge and mortar are presented. The tests revealed that replacement of 6% of cement with tannery sludge in the mix increased flexural and compressive strength and UPV values, whereas water absorption decreased. SEM and XRD analysis revealed that specimens with tannery sludge contained lower amounts of ettringite and higher amounts of portlandite; the obtained structure was denser and contained more calcium hydrosilicates (C-S-H). Chromium leaching values in cement mortars were found not to exceed the limit values set forth in Directive 2003/33/EC.

## 1. Introduction

Rational use of industrial waste is a major challenge in implementing the provisions of the Circular Economy Documentation and realising the concept of Cleaner Production when it comes to increasing production efficiency and reducing the risk to humans and the environment. Therefore, rational recycling of industrial waste is becoming a major challenge. As part of waste recycling and recovery programs, some natural materials will have to be replaced with waste in the future, including in the construction sector.

Volumes of tannery sludge (TS) are increasing all over the world. The sludge from leather processing consists of organic and inorganic substances. The chemical composition of inorganic substances commonly presented in tannery sludge includes the following elements and their chemical compounds: nitrogen, ammonia, sulphides, calcium compounds, chromium (III) salts (especially sulphates) as well as high chromium content [1,2,3,4,5,6,7]. The most toxic chemical element formed during leather tanning is chromium (VI) which is oxidized from chromium (III). Chromium (VI) is carcinogenic, mutagenic and can cause allergy. According to the current legislation [8,9], the permissible level of chromium in tannery sludge is 3 mg/kg. 

Scientists [10,11,12,13] claim that leather processing waste consists of 23% organic carbon, 9% calcium, 9% chromium and chlorides, sulphates and carbonates and the main component is calcite. Authors [14] additionally state that it can be made up of microorganisms that are hazardous to health. According to some research data [5], the sludge produced in the leather processing industry consists of chromium oxide, sodium chromium oxide, quartz, gypsum and chromium sulphate hydroxide. Experimental tests revealed [15,16] that when tannery sludge is added to cement, chromium oxide reacts with calcium ions and inhibits the formation of portlandite and C_3_S. In specimens with TS, more intense ettringite peaks and high levels of calcite are obtained. In addition, the leaching of chromium in cement specimens is very low. Research [17] has also shown that after mixing tannery sludge with different types of cement, the leaching of chromium does not exceed the maximum allowable limits specified in the relevant hygiene standards.

As has already been mentioned, one of the most harmful substances in leather processing is chromium, which is necessary to protect leather from water penetration and rotting. However, only 60–80% of the chromium is consumed in leather processing, the residual amount, when allowed by ecological standards, is removed through the sewer [18]. 

Harmful sludge in Europe normally cannot be disposed to landfills due to the high content of chromium that drains away from the materials and enters the soil and groundwater. Therefore, research is being carried out and attempts are being made to prepare this waste for proper use in the production of building materials. The results of research [19] show that the harmful effects of chromium are neutralized using TS waste in geopolymers. The addition of TS waste improves the compressive strength of the specimens. Reference [20] describes that the leaching of chromium is significantly reduced and bonded by the ladle furnace slag.

There are studies [21,22,23] which propose to neutralize chromium with activated carbon. Reference [24] discussed the possibility of removing 99% of chromium (III) from tannery sludge by using nanosized polymeric materials that have great potential to remove heavy metal cations from wastewater. Reference [24] presents the mineral composition of the sludge produced during leather processing. The investigated dried waste consisted of calcite (CaCO_3_) and quartz (SiO_2_) and at 1000 °C it also contained chromium (III) oxide and NaSO_4_. When used as an additive in cement mixes, this waste additive changes cement hydration, accelerates the initial and final setting time. A properly selected amount of waste can increase the compressive strength of cement. Some research proves an improvement in the strength of concrete if CaCO_3_ is added to concrete mixes. Other authors [25] report a healing of concrete cracks as a result of calcium instability in wet air. This phenomenon is beneficial for concrete structures. It is supposed that the said effect would be more useful in dry air conditions [26]. Scientists [27] have found that tannery sludge added at 0.7% by weight of cement does not change the chemical composition of cement but slightly reduces its flexural and compressive strength after 28 days. The waste investigated by these authors was made up of approximately 60% of organic combustible substances, which could have caused the strength to decrease. Researchers [28] have found that it would be possible to use such dried waste in cementitious materials to reduce the thermal conductivity of walls; however, the thermal conductivity starts increasing when the waste is moistened. 

The purpose of our work is to investigate the properties of dried TS waste and to determine its effect on the performance of cement mortars.

## 2. Materials and Methods 

The following materials were used for the specimens: cement CEM I 42.5 R (its chemical composition is presented in Table 1 and physical-mechanical properties in Table 2), sand as a fine aggregate (fraction 0/2), plasticising admixtures (superplasticizer Master Glenium sky 628), TS and water. 

Mineral composition of the cement: C_3_S—56.6%, C_2_S—16.7%, C_3_A—9.0%, C_4_AF—10.6% and 7.1% others (alkaline sulphates and CaO). 

The apparent density of sand established by applying the pycnometer method with isopropyl alcohol is equal to 2.64 g/cm^3^; the bulk density established by EN 1097-3:1998 is equal to 1.67 g/cm^3^, whereas the water absorption measured by EN 1097-6:2000 is equal to 0.65%. 

The amount of heavy metals in the eluate from cement specimens was determined by an atomic absorption spectral analysis method using a Buck Scientific 2010 VGP spectrometer (East Norwalk, CT, USA) with air-acetylene flame. The eluate was prepared according to standard LST EN 12457-2:2003, L/S equals to 0.1 l/kg; 3 samples of batch were tested.

Table 3 presents the results of the eluate test and extractable metals detected in the solid residue as well as other properties of the sludge. The results show that TS waste contains chlorides, sulphates, fluorides, and heavy metals. TS humidity reaches 78.4%, pH is 7.9, and total organic carbon is 0.66%. TS had a bulk density of 0.47 g/cm^3^, particle density of 1.67 g/cm^3^. Heavy metals present in TS do not exceed the limits specified in 2003/33/EC; however, chromium content is very close to the allowable limit values. 

**Figure 1:**
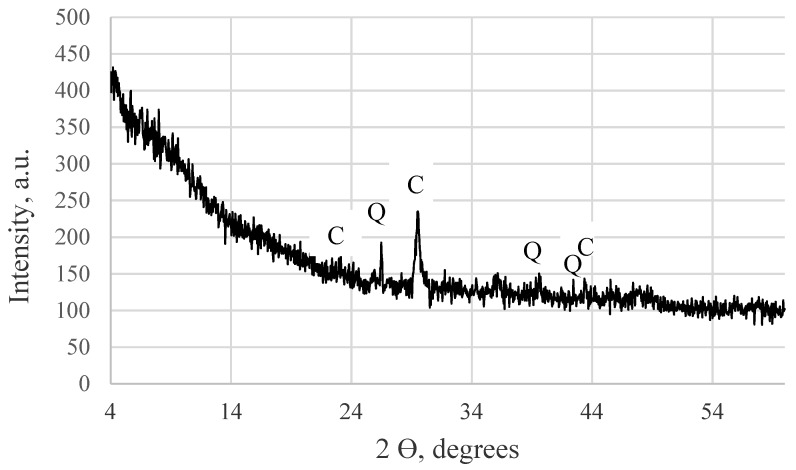
X-ray diffraction patterns of tannery sludge: C—calcite, Q—quartz.

Mineral composition tests of TS revealed that the TS mainly contains calcite and quartz minerals and a large amount of amorphous phase. Traces of chromium oxide and calcium sulphite hydrate were also detected (Figure 1).

**Figure 2:**
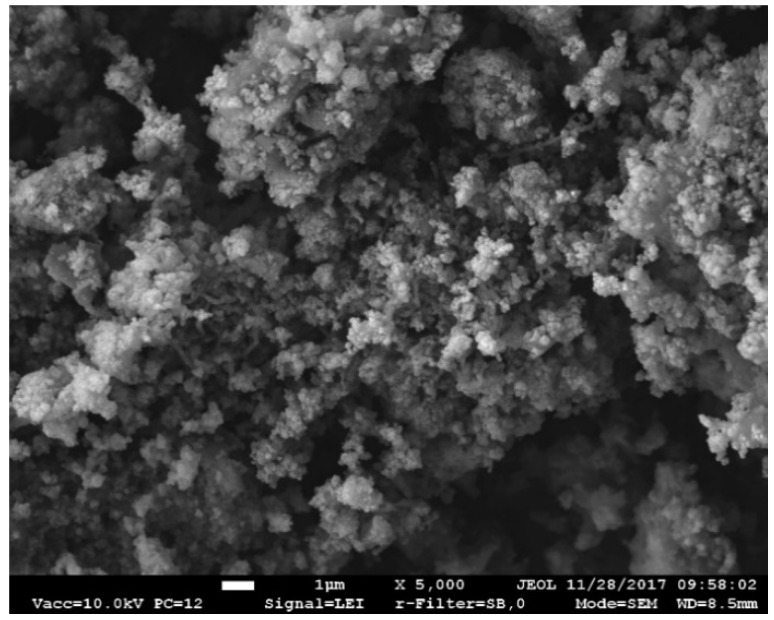
Scanning electron microscopy (SEM) image of tannery sludge (TS).

TS microstructural analysis revealed that the TS is amorphous and contains particles of different sizes and shapes. The particles are round, have a loose structure and an uneven surface. Smaller particles stick to larger particles, thus forming larger aggregates of particles (individual grains are clearly seen). The size of particle aggregates is ~0.10 µm (Figure 2). 

The superplasticizer complied with provisions of LST EN 934-2 and the water complied with the provisions of LST EN 1008:2003. The consistency of prepared mortars was determined according to LST EN 1015-3. The specimens were formed, cured and hardened in accordance with the standard LST EN 1015-11:2007. The compressive strength after 28 days (6 specimens) was measured by hydraulic press ALPHA3-3000S in accordance with the standard LST EN 1015-11:2007, sorptivity (6 specimens) was measured according to LST EN 1015-18. 

The microstructures of TS and mortars were observed with a scanning electron microscopy (SEM) device—JEOL JSM-7600F (Tokyo, Japan). The parameters of electron microscopy were as follows: voltage of 10 kV; distance to the surface of the specimen, from 7 to 10 mm. Prior to the test, the specimen surface was covered with a thin golden layer by gold electron vacuum evaporation.

X-ray diffraction (XRD) analysis of the phase composition of the materials was accomplished by diffractometer DRON-7 (Burevestnik, ST Petersburg, Russia). The following parameters were used for the tests: voltage—30 kV; current—12 mA; the range of the diffraction angle—from 4 to 60°, the detector movement step—0.02°; the duration of the intensity measuring in a step—0.5 s. Phase identification was carried out by decoding the XRD patterns according to ICDD diffraction databases.

The water absorption rate of cement mortars was determined by cutting the prisms (mortar specimens 160 mm × 40 mm × 40 mm, 6 specimens) into two equal parts, drying them to a constant mass and then weighting. Afterwards, the specimens were immersed in water and weighted after 10 min, 30 min, 60 min, 300 min, 24 h and 96 h.

The ultrasonic pulse velocity (UPV) of mortar prisms (3 specimens) was measured with the instrument Pundit 7 (transducer frequency 54 kHz, Proseq SA, Switzerland) and the velocity (V, m/s) was calculated from the equation:

V = l/τ(1)

where l is specimen length, m; τ is the time of transfer of ultrasonic signal through the specimen, s.

Ultrasonic pulse velocity is the characteristic of a material according to which changes of porosity and strength of materials can be estimated.

Two compositions of cement mortars were prepared: in composition C, a portion of the cement was replaced with chromium-rich tannery sludge; in composition S, a portion of sand was replaced with tannery sludge. Compositions were formulated, taking into consideration the findings of other researchers and previous work with similar waste [29,30] and thus using the maximum waste content up to 15%. Compositions of cement mortar mixes are presented in Table 4. Test compositions were prepared by mixing dry raw materials (powdered dry tannery sludge (dried at 105 °C ± 5 °C temperature was used) at first and then, adding water mixed with superplasticizer. 40 mm × 40 mm × 160 mm prisms were moulded from the compositions specified below. 

The analysis of extractable metals was done in accordance with LST EN 12457-2:2003, TS humidity was determined in accordance with LST EN 15934:2012. Cr^3+^ amount in the eluate was determined with an atomic absorption spectral analysis method using a Buck Scientific 2010 VGP spectrometer with air-acetylene flame. Eluate for chromium leaching tests was prepared in accordance with LST EN 12457-2:2003. Cr^3+^ leaching values were evaluated against the limit values specified in 2003/33/EC (2003/33/EC 2002) [9].

## 3. Results and Discussion

**Figure 3:**
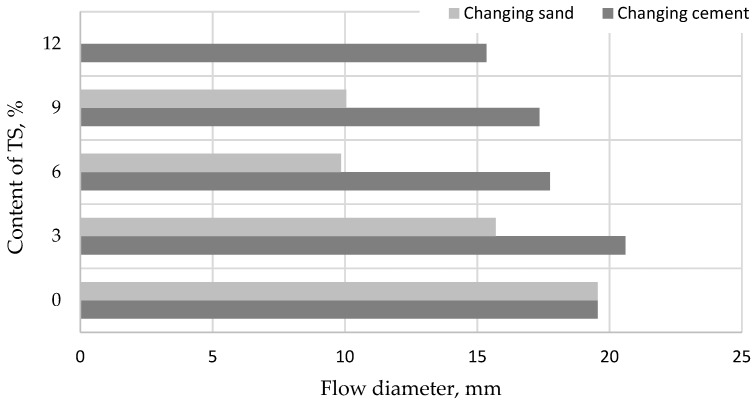
Relationship between mortar slump and TS content in the mix.

At first, the flowability of the mortars was tested. The flowability was found to decrease with a higher TS content in the mix (Figure 3). A sharper decrease in flowability was observed when part of the sand was replaced with TS. This effect can be explained by a higher content of TS in the mix compared to the replacement of the part of the cement. The higher porosity of the structure and significantly higher TS particle surface area, which requires more water to wet the particle, influenced the flowability decrease. It is not feasible to replace more than 9% of sand with TS because the workability of the mix deteriorates. When 9% of sand was replaced with TS, the moulding of specimens was aggravated, so the amounts of water and cement were increased and a constant ratio V/C = 0.5 was maintained. Nevertheless, the flowability was only 10 cm or 50% compared to the reference specimen. A less significant flowability decrease was observed when part of the cement was replaced with TS. With the maximum content of TS (12%), the flowability of the mix reduced by ~20%.

**Figure 4:**
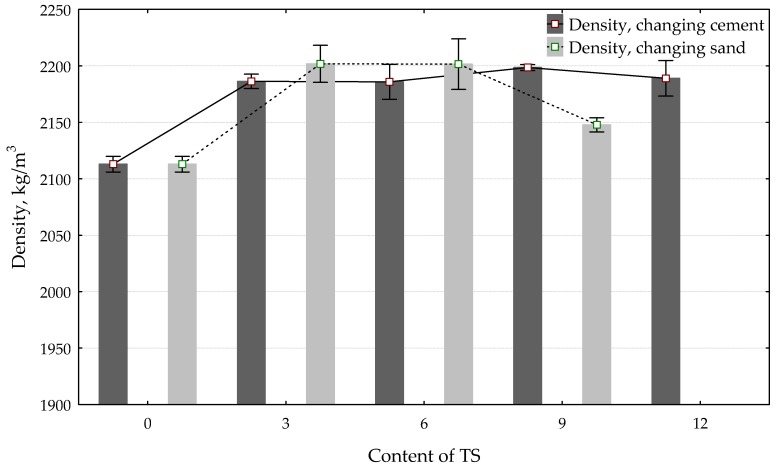
The impact of TS content in the mix on mortar density.

Figure 4 illustrates how the density of the mix depends on the amount of TS that replaces a part of sand or a part of the cement. 

**Figure 5:**
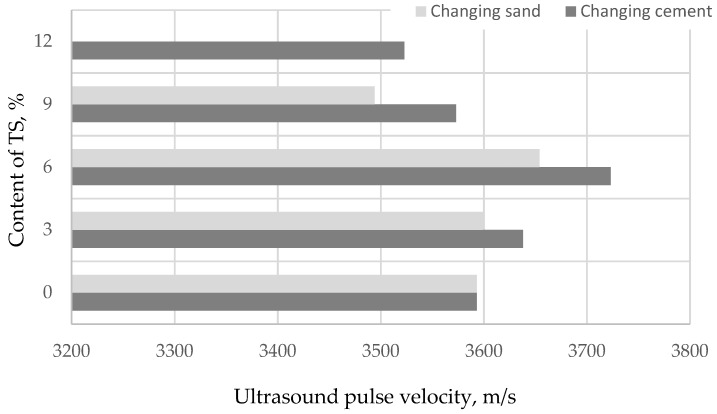
The impact of TS content on ultrasound pulse velocity (UPV).

The density of hardened mortar increases when up to 6% of the fine aggregate (sand) is replaced with TS (the density of a specimen containing 6% of TS increased by 7%); however, with further increase of TS content, the density of the hardened mortar starts decreasing. The increase in initial density is caused by the consumption of finer TS particles in the mix. These particles fill in the pores and act as crystallisation centres. During visual inspection, no pores were observed on the surface of specimens containing 6% of TS. The surface of the specimen is even and has a darker colour (Figure 5). The change in colour can be explained by the fineness of TS addition. The finer the addition, the more intensive the colour of the specimen. Similar changes in cement mortar colour were found by other researchers [31,32], who used waste materials in cement mixes. A decrease in the density of specimens containing TS > 7% occurs because higher TS content absorbs more water and reduces the water content required for the cement hydration process. Therefore, the mortar has more pores and capillaries, which in turn reduce the mortar density and subsequently the strength. 

In specimens with higher TS content, pores up to 1 mm in size were seen, as with the reference specimen. When up to 6% of cement is replaced with TS, a stable growth of ~4% in specimen density is observed. The bigger differences between density result when TS is used to replace a part of the cement or a part of the sand are explained by higher TS content in the mix calculated by the weight of the sand.

The change in density and structure of the specimens with higher TS content in the mix can be evaluated to some extent from the change of the ultrasonic pulse velocity (UPV) along the entire length of the specimen. The changes in UPV, depending on TS content in the mix, are presented in Figure 5.

Figure 5 illustrates that the highest UPV values are recorded in specimens where 6% of both sand and cement are replaced with TS. UPV values increase with the increase of TS amount in the mix up to 6%. UPV values decrease in mixes with a TS amount of above 6%. These specimens have more voids of different forms with entrained air. UPV increased ~4% in specimens with 6% of TS replacing a part of cement and in specimens with 6% of TS replacing a part of sand, the UPV increased by ~2%. Ultrasonic pulse velocity values make it possible to predict that cement mortars with 6% TS will have the highest strength.

**Figure 6:**
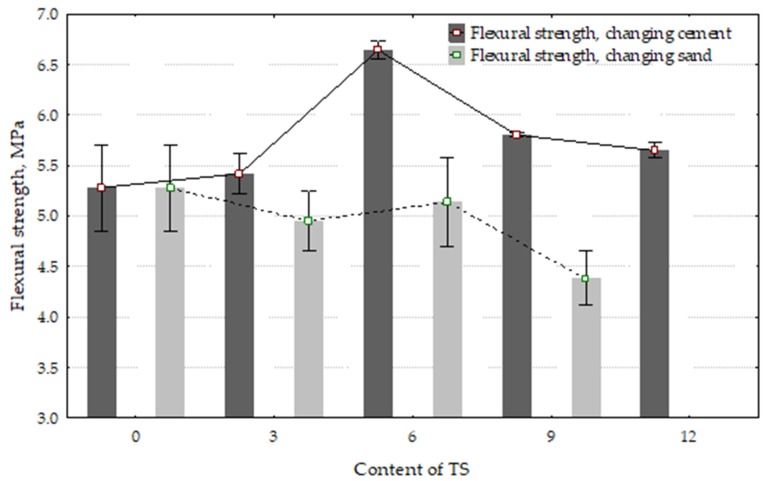
The effect of TS content in the mix on flexural strength.

Higher flexural strength was observed in all specimens containing up to 12% of TS in the cement mix compared to the reference specimen without TS (Figure 6). The highest flexural strength was observed in specimens containing 6% of TS. In this case, the strength increased 26%. Almost no changes in flexural strength were observed in specimens where up to 6% of the sand was replaced with TS; a reduction tolerance of 2% was observed. 

**Figure 7:**
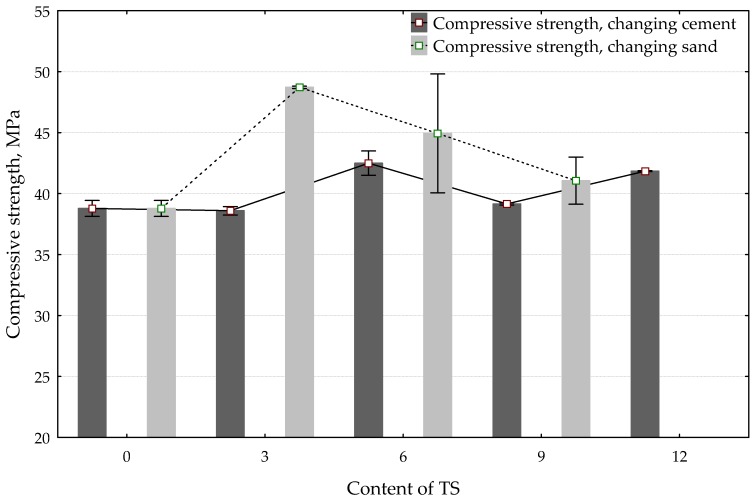
The effect of TS content in the mix on compressive strength.

Figure 7 illustrates the compressive strength results. The best compressive strength results were obtained in specimens where 3% of sand was replaced with TS. The compressive strength increased by 26%. With a higher content of TS in the mix, the compressive strength started decreasing; however, it remained higher, as compared to the reference specimen. In specimens where a part of the cement was replaced with TS, the best results were obtained in specimens containing 6% of TS. In such specimens, the compressive strength increased by 10%. 

The increase of strength was influenced by the pozzolanic properties of TS. The strength of the specimens increased even with lower cement content because TS particles acted as crystallisation centres. At first, they absorbed the majority of water, which was later released into the system and thus promoted hydration. Researchers [17] found that cement hydration rate depended on the chromium content in tannery sludge. Chromium present in tannery sludge is known to significantly increase cement hydration because of decreased viscosity of the gels formed and subsequently a greater diffusion rate due to the formation of nucleation points from which hydrate crystals grow up. This phenomenon is a function of TS percentage added. Authors found similar trends of compressive strength changes after 28 days of curing. Compressive strength gradually increased up to a certain amount of TS in the mix and started decreasing after a certain limit was reached.

**Figure 8:**
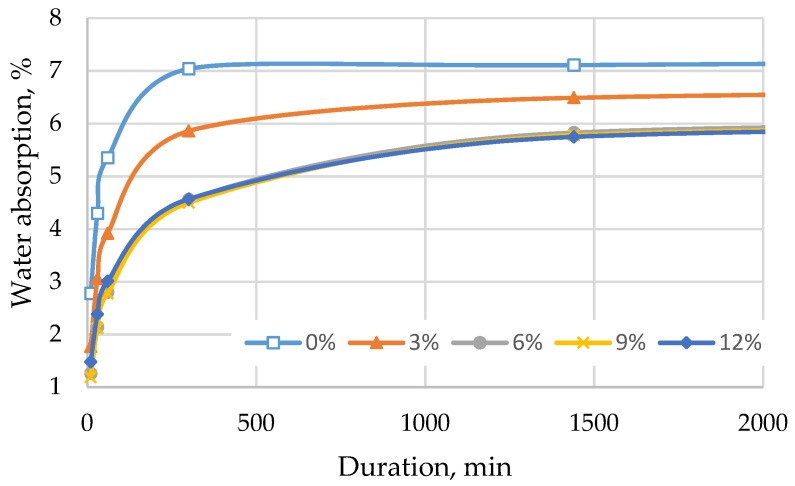
Water absorption when a part of cement is replaced with TS.

**Figure 9:**
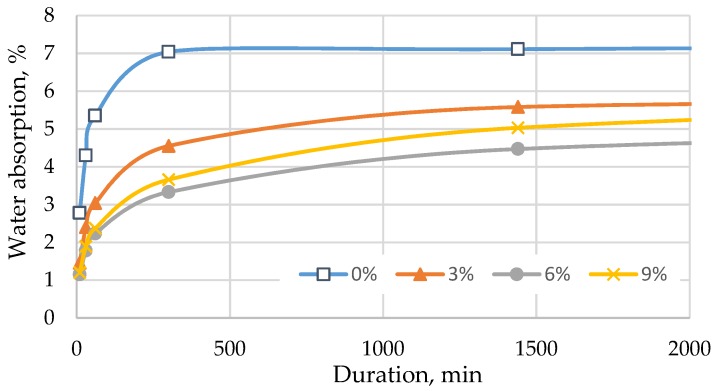
Water absorption when a part of sand is replaced with TS.

**Figure 10:**
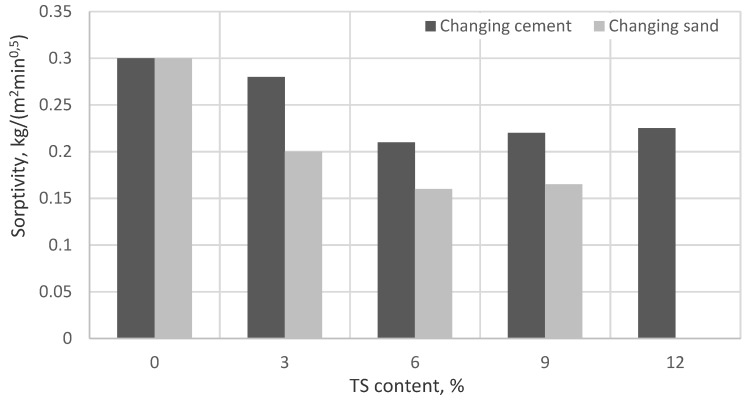
The impact of TS content on sorptivity.

**Figure 11:**
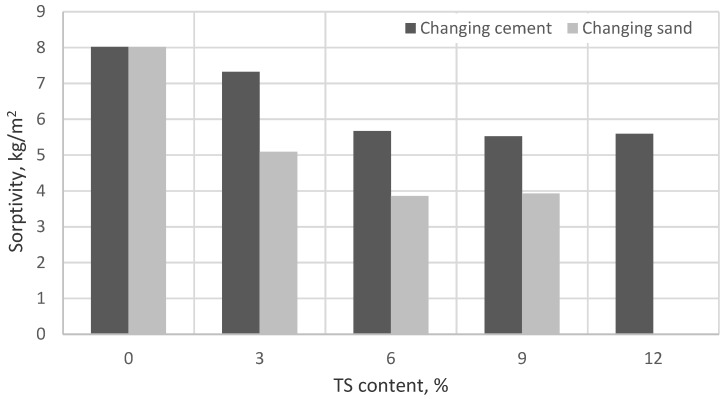
The impact of TS on the sorptivity in mortars used for renovation application.

Afterwards, the ability of cement mortar to absorb water was tested in two ways: by immersing the specimens completely in water (Figure 8 and Figure 9) and by immersing the cut edge of the specimen in water to a depth of 5–10 mm (Figure 10 and Figure 11).

Figure 8 illustrates that the water absorption of the mix decreases with a higher content of TS in the mix. In all batches, the standard deviation of water absorption was from 0.01% till 0.15%. Water absorption characteristics are usable for evaluating the frost resistance of mortars in external applications. Mortars with lower water absorption tend to decompose less when exposed to freezing and thawing cycles. The lowest water absorption was observed in specimens where 6% of cement was replaced with TS. In this case, water absorption decreased ~17% after 96 h of soaking. With the increase of TS content up to 12%, water absorption remained the same as in specimens with 6% of TS.

The trends of water absorption over time were similar in all cases: the specimens absorbed the greatest amount of water during the first 300 min; afterwards, the specimen became saturated and stopped absorbing water. In specimens where part of the sand was replaced with TS, the best water absorption results were observed in specimens containing 6% of TS. After 24 h, water absorption reduced by 37% and by 28% after 96 h. Specimens containing TS absorbed water slower, as compared to specimens without TS. This test also confirmed the observation that it is not feasible to replace more than 6% of sand with TS because of changes in the structure. 

The sorptivity values are presented in Figure 10 and Figure 11.

The values of sorptivity presented in Figure 10 were determined for ordinary mortars when the results were recorded 10 and 90 min after the specimens had been saturated with water due to capillary action. The average standard deviation of all batches was 0.01 kg/m^2^min^0.5^. Values of sorptivity presented in Figure 11 were determined by measuring the initial mass of the specimen and the mass after saturation due to capillary action after 24 h. The average standard deviation of all batches was 0.14 kg/m^2^. The results obtained in both tests were very similar. The lowest sorptivity was obtained in mortars containing 6% of TS. Sorptivity in specimens where 6% of the cement was replaced with TS reduced by ~30%, as compared to the reference specimen and in specimens where 6% of the sand was replaced with TS, sorptivity reduced by 50%. Besides, those specimens containing 6% of TS had the lowest surface porosity. It can be explained by the optimal amount of TS and the water amount used. TS has a rather large specific surface area through which water required for cement hydration is easily absorbed. Fine TS particles saturated with water fill in the voids between cement particles and thus promote a faster release of water to C_3_S crystals where C-S-H formation starts. A cement matrix with a dense structure is formed. When the TS amount in the mix is increased, there is not enough water to wet TS particles and thus cement binding decelerates and the porosity of the mortar increases.

**Figure 12:**
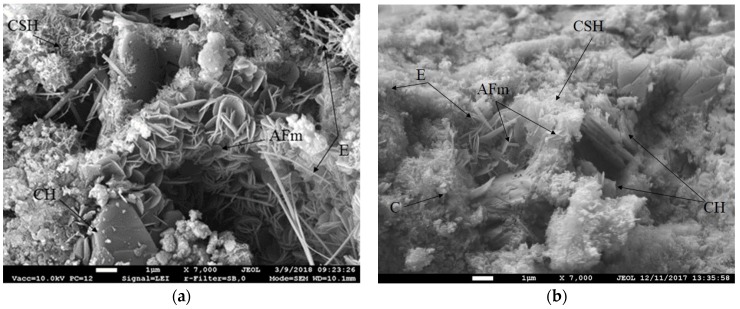
Microstructure images of (**a**) specimen without TS, and (**b**) specimen with 6% of sand replaced with TS (magnification: ×7000).

The best results were obtained with specimens in which 6% of the sand was replaced with TS; therefore, SEM analysis of these specimens and reference specimens was performed. The SEM images are presented in Figure 12.

Both images illustrate portlandite plates (CH) growing as well as needle-shaped ettringite (E) growing in the voids. In addition, they show calcium silicate hydrate (C-S-H) in the form of comb and lattice framework and AFm in the form of fine plates developing in cavities when there is much CH and ettringite in the matrix. According to Reference [33], tiny AFm plates grow in larger pores and divide large pores into small pores as a frame, which causes the most probable aperture to become small. On the other hand, the thin plates promote the formation of C-S-H, the size of which decreases due to the limited growth space. The C-S-H fills the space between the thin plates and creates a network structure to reduce the porosity. Specimens of this batch had the lowest porosity and one of the highest strengths (Figure 12b). The images also illustrate that the structure of mortar containing 6% of TS is denser than the structure of the reference specimen. No open pores are visible, the average pore size is ~2 µm, crystals are smaller; there is a higher amount of C-S-H and a very low amount of ettringite. The experimental results show that a finer TS leads to faster cement hydration, lower amounts of ettringite and higher amounts of C-S-H create a higher strength of mortar. Calcite (C) is visible more clearly in specimens with TS. 

**Figure 13:**
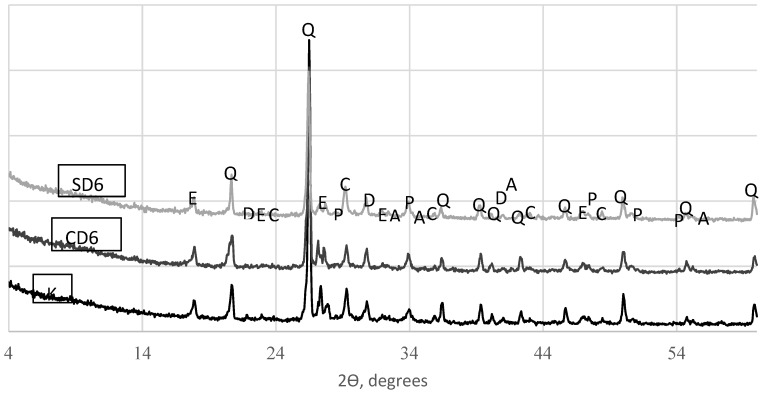
X-ray diffraction (XRD) patterns (E—ettringite, Q—quartz, D—dolomite, C—calcite, P—portlandite, A—alite).

X-ray diffraction analysis was performed for specimens containing 6% of TS and reference specimens to find what type of minerals had formed in the cement mortar. X-ray diffraction patterns are presented in Figure 13. 

X-ray diffraction patterns revealed the same minerals, only in different amounts, in all mixes—portlandite (Ca(OH)_2_), quartz (SiO_2_), ettringite (3CaO·Al_2_O_3_·3CaSO_4_·31H_2_O), calcite (CaCO_3_) dolomite (CaMg(CO_3_)_2_, and alite (3CaO·SiO_2_). Figure 13 illustrates that the specimen with 6% TS contained lower amounts of ettringite and alite and higher amounts of portlandite. Portlandite does not have a significant effect on the strength of the structure, however a higher content of the mineral creates a pH of 12.5–13 necessary for smooth cement hydration [34,35]. This means that the amount of hydration had increased in specimens with TS, thus leading to the increase in specimen strength and cement hydration acceleration.

It must be ensured that the leaching of chromium will not exceed the limit values in order to use tannery sludge in cement mortar manufacture. According to Directive 2003/33/EC, chromium leaching from non-hazardous waste may not exceed 2.5 mg/L. 

Tests of chromium leaching from cement mortars revealed that even with the highest tannery sludge addition of 9%, the amount of leached chromium does not exceed the limit values of non-hazardous waste (2.5 mg/L) according to the normative document (Table 5). 

The average standard deviation of chromium leaching in all batches was 0.01 mg/l. Therefore, it can be stated that tannery sludge can be safely recycled in cement mortar manufacture. However, chromium leaching increases in mortar when the content of TS increases. After adding 9% TS, the chromium leaching, compared to the control sample, increases by 58% and it is close to the requirements (<0.2 mg/L). We recommend using a composition with 6% TS, the chromium leaching of which is 0.17.

## 4. Conclusions

It was found that it is feasible to use TS in construction mortars by replacing 6% of cement or natural aggregate—sand—with TS. Up to a 6% addition of TS reduced porosity and water absorption of the mortar, increased the strength up to 26% and increased density and ultrasonic pulse velocity by ~4%. In this way, the expensive binding material—cement—can be saved. 

XRD analysis showed that all analysed mortars contained similar standard minerals—portlandite, quartz, ettringite, calcite, dolomite, and alite. Specimens modified with TS contained a higher amount of portlandite, C-S-H and a lower amount of ettringite, as well as a lower amount of non-hydrated cement minerals. XRD analysis results were confirmed by SEM tests. 

The values of chromium leaching from cement mortars met the requirements for non-hazardous waste prescribed in 2003/33/EC and in the normative documents of other countries. 

## Figures and Tables

**Table 1:** Chemical composition of cement.

CaO	SiO_2_	Al_2_O_3_	Fe_2_O_3_	MgO	K_2_O	Na_2_O	SO_3_	Cl	L.O.I.
63.2	20.4	4.0	3.6	2.4	0.9	0.2	3.1	0.05	2.15

**Table 2:** Physical-mechanical properties of cement.

Particle Density, g/cm^3^	Soundness “Le Chatelier”, mm	Passing 32 µm, %	Compressive Strength after 2 Days, MPa	Compressive Strength after 28 Days, MPa	Vicat Initial, min	H_2_O, %
3.1	1.0	78.5	30	55	180	27.5

**Table 3:** Analysis of extractable metals in the solid residue and other properties of tannery sludge.

Parameter	Results
pH	7.9
Dry matter, %	78.4
Chlorides, mg/kg	3688
Sulphates, mg/kg	6288
Fluorides, mg/kg	0.80
Cadmium, mg/kg	<0.001
Lead, mg/kg	<0.001
Chromium, mg/kg	0.416
Nickel, mg/kg	<0.052
Copper, mg/kg	0.208
Zinc, mg/kg	0.544
Barium, mg/kg	0.152
Selenium, mg/kg	<0.001
Stibium, mg/kg	<0.001
Arsenic, mg/kg	0.024
Mercury, mg/kg	0.001
Dry residue, mg/kg	15728
Molybdenum, mg/kg	0.024
Dissolved organic carbon, mg/kg	42.4
Total organic carbon, %	0.66

**Table 4:** Compositions of cement mortar mixes.

Designation:	Compositions of Cement Mortar Mixes Per One Cubic Meter					
	Tannery Sludge, %	Tannery Sludge, g	Cement Content, g	Water Content, g	Superplas-Ticizer, g	Sand 0/2, g
K	0	0	500	250	5	1500
Replacing a Part of Cement						
CD3	3	15	485	250	5	1500
CD6	6	30	470	250	5	1500
CD9	9	45	455	250	5	1500
CD12	12	60	440	250	5	1500
Replacing a Part of Sand						
SD3	3	45	500	250	5	1455
SD6	6	90	500	250	5	1410
SD9	9	135	600	300	5	1365

**Table 5:** Tests in chromium leaching from cement mortars.

Parameters	Cement Mortars			
	K	SD3	SD6	SD9
Cr, mg/L	0.12	0.14	0.17	0.19

## References

[1] Szpryokowicz L., Grandi Z.F. (1995). Electrochemical treatment of tannery wastewater using Ti/Pt and Ti/Pt/Ir electrodes. Water Res..

[2] Naumczyk J., Szpyrkowicz L., De Faveri D.M., Grandi Z.F. (1996). Electrochemical treatment of tannery wastewater containing high strength pollutants. Trans. Inst. Chem. Eng..

[3] Sykes R.U., Corning D.R. (1987). Pollution Abatement and Control in Leather Industry, Industry and Environment.

[4] Vlyssides A.G., Israilides C.J. (1997). Detoxification of tannery waste liquors with an Electrolysis system. Environ. Pollut..

[5] Pinto A.C., Valenzuela-Diaz F.R., Sansalone J.J., Dweck J., Cartledge F.K., Büchler P.M. (2006). X-Ray Diffraction study of particulate tannery waste solidified in cement. Mater. Sci. Forum.

[6] Houshyar Z., Khoshfetrat A.B., Fatehifar E. (2012). Influence of ozonization process on characteristics of pre-alkalized tannery effluents. Chem. Eng. J..

[7] Torras J., Buj I., Rovira M., de Pablo J. (2012). Chromium recovery from exhausted baths generated in plating processes and its reuse in the tanning industry. J. Hazard. Mater..

[8] Hu J., Xiao Z., Zhou R., Deng W., Wang M., Ma S. (2011). Ecological utilization of leather tannery waste with circular economy model. J. Clean. Prod..

[9] (2003). Council Decision of 19 December 2002 Establishing Criteria and Procedures for the Acceptance of Waste at Landfills Pursuant to Article 16 of and Annex II to Directive 1999/31/EC (2003/33/EC).

[10] Shen D., Huang M., Feng H., Li N., Zhou Y., Long Y. (2017). Effect of waste addition points on the chromium leachability of cement produced by co-processing of tannery sludge. Waste Manag..

[11] Kavouras P., Pantazopoulou E., Varitis S., Vourlias G., Chrissafis K., Dimitrakopulos G.P., Mitrakas M., Zouboulis A.I., Karakostas T., Xenidis A. (2015). Incineration of tannery sludge under oxic and anoxic conditions: Study of chromium speciation. J. Hazard. Mater..

[12] Pantazopoulou E., Zouboulis A. (2018). Chemical toxicity and ecotoxicity evaluation of tannery sludge stabilized with ladle furnace slag. J. Environ. Manag..

[13] Pantazopoulou E., Zebiliadou O., Mitrakas M., Zouboulis A. (2017). Stabilization of tannery sludge by co-treatment with aluminum anodizing sludge and phytotoxicity of end-products. Waste Manag..

[14] Ping T., Zhao Y., Xia F. (2008). Thermal behaviors and heavy metal vaporization of phosphatized tannery sludge in incineration process. J. Environ. Sci..

[15] Barros A.M., Soares Ten’orio J.A. Effect of Cr_2_O_3_ in the formation of clinker of Portland cement. Proceedings of the Memorias 5th Congreso Brasileiro de Cimento.

[16] Stephan D., Maleki H., Knofel D., Eber B., Hardtl R. (1999). Influence of Cr, Ni and Zn on the properties of pure clinker phases: Part I. C3S. Cem. Concr. Res..

[17] Montañés M.T., Sánchez-Tovar R., Roux M.S. (2014). The effectiveness of the stabilization/solidification process on theleachability and toxicity of the tannery sludge chromium. J. Environ. Manag..

[18] Kowalski Z. (1994). Treatment of chromic tannery wastes. J. Hazard. Mater..

[19] Jin M., Lian F., Xia R., Wang Z. (2018). Formulation and durability of a geopolymer based on metakaolin/tannery sludge. Waste Manag..

[20] Alaerts G.I., Jitjarurunt V., Kelderman P. (1989). Use of coconut shell based activated carbon for chromium(VI) removal. Water Sci. Technol..

[21] Ramos L.R., Jurez Martinez A., Gruerrero Coronado R.M. (1994). Adsorption of chromium(VI) from aqueous solutions on activated carbon. Water Sci. Technol..

[22] Fahim N.F., Barsoum B.N., Eid A.E., Khalil M.S. (2006). Removal of chromium (III) from tannery waste water using activated carbon from sugar industrial waste. J. Hazard. Mater..

[23] El-Sherif I.Y., Tolani S., Ofosu K., Mohameda O.A., Wanekay A.K. (2013). Polymeric nanofibers for the removal of Cr (III) from tannery waste water. J. Environ. Manag..

[24] Trezza M.A., Scian A.N. (2007). Waste with chrome in the Portland cement clinker production. J. Hazard. Mater..

[25] Madrid M., Orbe A., Rojí E., Cuadrado J. (2017). The effects of by-products incorporated in low-strength concrete for concrete masonry units. Constr. Build. Mater..

[26] Ranaivomanana H., Verdier J., Sellier A., Bourbon X. (2013). Sealing process induced by carbonation of localized cracks in cementitious materials. Cem. Concr. Compos..

[27] Piasta W. (2017). Analysis of carbonate and sulphate attack on concrete structures. Eng. Fail. Anal..

[28] Lakrafli H., Tahiri S., Albizane A., Bouhria M., El Otmani M.E. (2013). Experimental study of thermal conductivity of leather and carpentry wastes. Constr. Build. Mater..

[29] Chen H., Ma X., Dai H. (2010). Reuse of water purification sludge as raw material in cement production. Cem. Concr. Comp..

[30] Malaiskiene J., Kizinievic O., Kizinievic V., Boris R. (2018). The impact of primary sludge from paper industry on the properties of hardened cement paste and mortar. Constr. Build. Mater..

[31] Kappel A., Ottosen L.M., Kirkelund G.M. (2017). Colour, compressive strength and workability of mortars with an iron rich sewage sludge ash. Constr. Build. Mater..

[32] Kappel A., Ottosen L.M., Kirkelund G.M., Goltermann P., Bache A.M. The colour potentials of SSA-containing mortar. Proceedings of the Concrete—Innovation and Design.

[33] Ma S., Li W., Zhang S., Hu Y., Shen X. (2015). Study on the hydration and microstructure of Portland cement containing diethanol-isopropanolamine. Cem. Concr. Res..

[34] Brykov A.S., Hydration of Portlandcement SPbGTI(TU). https://docplayer.ru/69799866-Gidrataciya-portlandcementa.html.

[35] Brykov A.S., Vasil’ev A.S., Mokeev M.V. (2013). Hydration of Portland Cement in the Presence of Aluminum-Containing Setting Accelerators. Russian J. Appl. Chem..

